# Recent Advances in Quenchbody, a Fluorescent Immunosensor

**DOI:** 10.3390/s21041223

**Published:** 2021-02-09

**Authors:** Jinhua Dong, Hiroshi Ueda

**Affiliations:** 1 Tokyo Tech World Research Hub Initiative (WRHI), Institute of Innovative Research, Tokyo Institute of Technology, Yokohama 226-8503, Japan; jdong@pe.res.titech.ac.jp; 2 Laboratory for Chemistry and Life Science, Institute of Innovative Research, Tokyo Institute of Technology, Yokohama 226-8503, Japan; 3 School of Life Science and Technology, Weifang Medical University, Weifang 261053, China

**Keywords:** antibody, immunoassay, fluorescence, quench, detection, biomarker

## Abstract

The detection of viruses, disease biomarkers, physiologically active substances, drugs, and chemicals is of great significance in many areas of our lives. Immunodetection technology is based on the specificity and affinity of antigen–antibody reactions. Compared with other analytical methods such as liquid chromatography coupled with mass spectrometry, which requires a large and expensive instrument, immunodetection has the advantages of simplicity and good selectivity and is thus widely used in disease diagnosis and food/environmental monitoring. Quenchbody (Q-body), a new type of fluorescent immunosensor, is an antibody fragment labeled with fluorescent dyes. When the Q-body binds to its antigen, the fluorescence intensity increases. The detection of antigens by changes in fluorescence intensity is simple, easy to operate, and highly sensitive. This review comprehensively discusses the principle, construction, application, and current progress related to Q-bodies.

## 1. Introduction

The detection of viruses, disease markers, physiologically active substances, drugs, and chemicals is important for many areas of our lives. Immunoassays are based on the specificity and affinity of antigen–antibody reactions. Nowadays, many antibodies against not only proteins but also small molecules (peptides and haptens) with high specificity and affinity can be obtained. As an analytical methodology for such small molecules, liquid/gas chromatography coupled with mass spectrometry (LC/MS and GC/MS, respectively) are frequently used as comprehensive analytical methods. However, compared with LC/MS or GC/MS, which require large and costly equipment and have a relatively long analysis time, immunoassays have the advantages of simple operation, shorter analysis time and good sensitivity as well as selectivity of detection. They are thus widely used in disease diagnosis and food/environmental monitoring.

Irrespective of target size, there are two kinds of immunoassays based on their operation mode: homogeneous and heterogeneous assays. Heterogeneous immunoassays need separation of the solid and liquid phases for separating antigen-bound and free (B/F) antibodies; representative detection technologies include enzyme-linked immunosorbent assay (ELISA) [1], immunofluorescence detection, and so on. Because of the many operation steps and long incubation time for reactions, these assays consume a lot of labor and time. In contrast, homogeneous immunoassays do not require the B/F separation and have the advantage of simple operation. However, we need an alternative way to distinguish the antigen-bound and free antibodies to realize it.

Quantitative analysis of protein location and concentration is key to understand their in situ functions. In principle, scaffolds combined with environment-sensitive fluorophores can detect analytes of interest with high temporal and spatial resolution. However, their adoption can be limited due to the extensive experimental screening required for their development. Wittrup’s group recently described their trials to generalize design principle of such “Scaffold conjugated to environment-sensitive fluorophore (SuCESsFul) biosensors” based on various binding protein scaffolds, analytes and fluorescent dyes [2]. After making more than 400 biosensors, they found that the brightest reading can be obtained when a specific binding pocket for the fluorophore is present on the analyte. They also found that the interaction between the fluorophore and binding protein can raise the background fluorescence and limit the dynamic range of the sensor. Islam et al. designed a wavelength-dependent fluorescent immunosensor by integrating a polarity indicator (3-(6-acetylnaphthalen-2-ylamino)-2-aminopropanoic acid, Anap) into the specific position of the anti-epidermal growth factor receptor (EGFR) single-chain variable region (scFv) to generate an emission-wavelength-related immunosensor. They found that when binding in the topological neighborhood of the antigen-binding interface, EGFR can titrate the blue shift of the emission wavelength of Anap, and the maximum wavelength can reach 20 nm, which has a nanomolar detection limit [3]. It is worth noting that these environment-sensitive dye-based approaches are considered effective only for larger protein targets that endow sufficient binding surface near the labeled dye to get distinct signals, and not for smaller molecules such as haptens and peptides.

Some years ago, Ueda and colleagues reported another type of biosensor, Quenchbody (Q-body), which is an immunosensor that can perform noncompetitive homogeneous assays for various antigens including small molecules. The key point of this technology is to label antibodies with fluorescent dye(s) at specific position(s); when the antibody fragments bind to the antigen, their fluorescence intensity increases and this change in fluorescence intensity is used to detect the antigens [4,5]. In this review, in addition to the brief introduction, we will describe recent applications and advances of Q-body technology.

## 2. The Working Mechanism and Types of Q-Bodies

**Figure 1:**
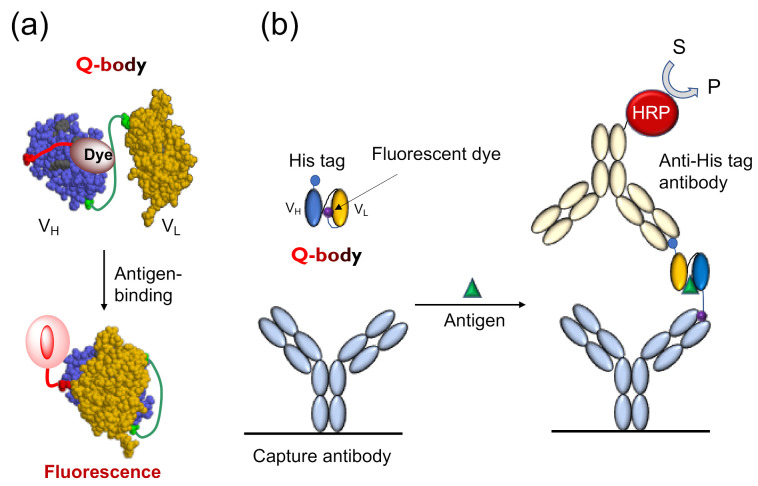
The working mechanism of Quenchbody. (**a**) The working model of Quenchbody. Trp residues are shown in light green.; (**b**) An ELISA to demonstrate the movement of the fluorescent dye on Quenchbody [5].

The Q-body is an antibody-based immunosensor, most frequently constructed by labeling the *N*-terminal region of scFv or the antigen-binding fragment (Fab) of an antibody with a fluorescent dye. The structure and working principle of a Q-body are shown in Figure 1a. When a specific fluorescent dye such as tetramethylrhodamine (TAMRA), rhodamine 6G (R6G), and ATTO520 is attached near the *N*-terminus of an antibody with a short (4~25 aa) flexible linker peptide, the fluorescent dye enters the variable regions of the antibody (Fv; V_H_ + V_L_) due to the dye’s hydrophobicity. Specific amino acids, namely, several tryptophan (Trp) residues in the antibody variable region weaken the fluorescence of the dye by fluorescence quenching due to photoinduced electron transfer (PET) from Trp to the dye [4]. In general, when the fluorescent dye is illuminated with the excitation light, the electrons transition from a low-energy orbit to a high-energy orbit, and emit fluorescence when these return to their low-energy orbit. However, in the vicinity of an amino acid that can provide electrons, the fluorescent dye takes electrons from the amino acid and does not emit fluorescence, termed as fluorescence quenching. However, when the Q-body binds to an antigen, due to antigen-dependent Fv stabilization and steric hindrance due to bound antigen, the quenched dye can no longer dwell in the interior of the antibody and moves to the exterior, and recovers its fluorescence. The antigen concentration can thus be detected by measuring the positive change in fluorescence intensity. This technique is easy to operate by simply adding the sample to the probe solution, by measuring the fluorescence intensity in a few seconds to minutes.

Tryptophan endowed with an indole side chain is an effective electron donor in the reaction with dye molecules, as it is one of the most easily oxidized functional groups among natural amino acids. Antibody fragments quench the coupled fluorescent dye through the Trp residue semiconserved in its variable region, as shown in Figure 1a. There are four conserved (>94%) Trp residues in an Fv region (36H, 47H, 103H, and 35L, according to Kabat numbering scheme [6]), and many antibodies have more in their complementarity determining region (CDR). In the absence of antigens, fluorescent groups enter the interface of the variable region of the heavy chain (V_H_) and variable region of the light chain (V_L_), and interact with Trp residues either directly through hydrophobic and π-π stacking interactions, or indirectly through the protein. It is known that such electron tunneling can happen effectively within 1 nm distance, as exemplified in the photosynthetic reaction center [7]. Hence, the position of the dye that causes optimal quenching is not very easy to predict even if the positions of the Trp residue are known. However, it is worth mentioning that the two conserved core Trp residues (36H and 35L) play a significant role in quenching [4], showing the potential generality of Q-body principle.

To explore the antigen-dependent characteristics of the Q-body and the intramolecular mechanism of its action, Ohashi et al. analyzed the dynamics of dye movement in the reaction between the Q-body and antigen by ELISA as well as fluorescence polarization techniques (FP) [8]. When they analyzed the available TAMRA dye attached to the Q-body against osteocalcin (BGP), as shown in Figure 1b, the more fluorescent dyes moved from the inside of the antibody to the outside, the more easily they were captured by the anti-dye antibody. The ELISA results showed that the signal in the presence of the antigen osteocalcin peptide (BGP-C7) was significantly higher than that in the absence of the antigen. Therefore, they carried out further experiments to detect the differences in signals in the presence of different antigen concentrations. As expected, the results were consistent with the fluorescent intensity detection. The more the antigen, the more fluorescent dyes were detected. This also explains why the fluorescence intensity increases with increasing antigen concentration. The effect of the linker length between the antibody and dye on the dye movement was also analyzed. The results indicated that a longer linker resulted in a stronger signal, as in the case of Q-body response. To confirm this conclusion, an experiment with an anti-bisphenol A (BPA) antibody was also conducted, and similar results were obtained. The FP results used to analyze the molecular tumbling of fluorescent dye indicated that the antigen concentration- and linker length-dependencies were also consistent with the ELISA results. The higher the antigen concentration, the more fluorescent dyes were released to the outside of the antibody; thus, the Brownian motion rate of the dye molecules increased and the degree of polarization decreased.

The ELISA and FP results indicated that enhancement of fluorescence intensity was caused by the outward movement of fluorescent dyes in the molecule, and the same results were also obtained for other two Fab-type Q-bodies for small molecular weight antigens, namely, methamphetamine analog and deoxynivalenol. Hence, the primary working mechanism of the Q-bodies for small molecules (haptens or peptides) will be summarized as the intramolecular competition between the antibody-tethered dye and the antigen in a sample. For the Q-bodies for protein antigens, another quenching mechanism also plays an important role, which will be explained later.

## 3. Design and Construction of the Q-Body

**Figure 2:**
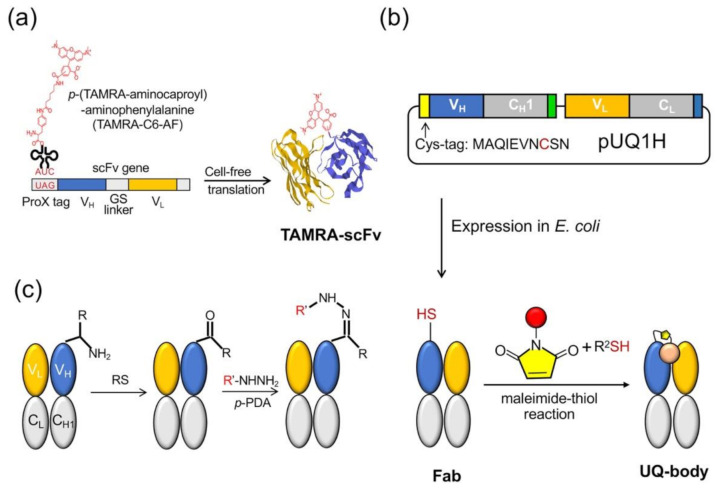
Quenchbody preparation. (**a**) Cell-free system for the scFv type. (**b**) Post labeling method to prepare an ultra-Q-body (UQ-body) with antigen-binding fragment (Fab) produced in *E. coli*. (**c**) Method based on transamination at the *N*-terminus. RS: Rapoport’s salt; *p*-PDA: *p*-phenylenediamine.

The Q-body was first discovered accidentally when scFv was specifically labeled with fluorescent dye-conjugated aminoacyl tRNA using cell-free translation technology [9]. The early Q-bodies were mainly designed based on the scFv or Fab format and synthesized by cell-free translation technology (Figure 2a). Abe et al. later reported that Fab fragments can be expressed by prokaryotic cells, and that the reaction of cysteine and maleimide can be used to label antibody fragments for preparing an ultra-Q-body (UQ-body) (Figure 2b). The UQ-body is more stable and generally gives higher sensitivity than the scFv type Q-body, probably due to deeper quenching [10].

The preparation of the Q-body either uses a cell-free translation system or a combination of recombinant *Escherichia coli* expression and subsequent fluorescent dye labeling. In a cell-free system, the fluorescent dye is usually introduced to the *N*-terminal region of an antibody fragment as a kind of unnatural amino acid [11]. However, in spite of quantitative incorporation, the limited amount of protein obtained by this method leads to an increase in production costs. Another method is to combine the expression of antibody fragments by *E. coli* and labeling them with the fluorescent dye through the thiol-maleimide reaction. However, both these methods are time-consuming due to the necessity of vector construction, protein expression, and purification. Furthermore, the maleimide-thiol linkage has lower yield and is not so stable in reducing environment. A recent attempt by Kurumida et al. introduced an unnatural amino acid (UAA) 3-azido-L-tyrosine into an scFv that recognizes Nef protein, a gene product of human immunodeficiency virus (HIV) using *E. coli* harboring special tRNA and aminoacyl tRNA transferase. They made Cy3-labeled Q-bodies by the postlabeling of the azido group by click chemistry using dibenzocyclooctyne (DBCO)-dye [12]. Although the labeling efficiency was estimated as 20–50%, this method does not involve Cys, which might interfere with protein folding, and gives more chemically stable dye conjugates. After optimizing the UAA position, they attained the maximum change in fluorescence intensity of 2-fold.

Dong et al. developed another method to prepare Q-bodies based on transamination, which can simply label the full-length antibody and prepare the Q-body based on the full-length antibody [13]. Transamination is a chemical reaction between amino acids containing amino groups, and ketone acids containing ketone groups. In this reaction, the NH_2_ group on one molecule is exchanged with keto acid. Gilmore et al. reported a site-specific transamination reaction, which introduces a new ketone group at the nitrogen end of the protein and incubated it with pyridoxal 5′-phosphate [14]. The carbonyls introduced by this reaction are not naturally present in proteins; therefore, by forming hydrazone or stable oxime bonds, they are used as unique attachment points for synthetic groups [15]. Witus et al. reported an improved protein diamine reagent, n-methylpyridine-4-aldehydebenzenesulfonate (Rapoport’s salt), with low requirements for specific reaction conditions [16]. Dong et al. used two kinds of fluorescent probes, commercially available aminooxy-5(6)-carboxytetramethylrhodamine (TAMRA) and chemically synthesized TAMRA-C5-peptide-hydrazide, to prepare Q-bodies (Figure 2c). In a denaturation experiment using 7 M guanidine hydrochloride (GdnHCl), the fluorescence intensity of prepared Q-bodies increased to 1.95–3.5-fold. The limit of detection (LOD) for the antigen (osteocalcin C-terminal 7 residue peptide; or bone Gla protein, BGP-C7) prepared by transamination was 10 nM, which was similar to the value obtained by competitive ELISA using the same antibody fragment (20 nM) [17]. The *N*-terminal amino acid sequence is also an important factor in the transamination reaction. Some amino acid sequences, such as EES, MEE, and AKT, have been reported to be more suitable for this reaction, whereas others, such as AEE, may not be able to effectively modify the keto groups. In their study, Dong et al. expressed Fab and found that the *N*-terminal amino acid sequence of the Fd fragment (V_H_-C_H_1) was ATG, whereas that of the light chain was SDI. They found that ATG was a good transamination sequence whereas SDI was not. This finding will be useful for labeling antibodies as well as other proteins.

The dye labeling of the antibody is usually performed at the *N*-terminus, near the antigen-binding site, but does not interfere with the antigen binding activity of the Q-body. To date, researchers have used an in vitro translation system or posttranslational method to fuse the dye-containing peptide with the *N*-terminus of Fab or the scFv of the antibody, indicating the site-specific labeling of cysteine residues in the peptide tags. Bilgicer et al. reported the specific binding strategy of the antibody variable region using indole-3-butyric acid (IBA) cross-linked to a nucleotide-binding site (NBS) by ultraviolet radiation, without affecting the antibody structure and function [18]. Based on the above research, Jeong et al. developed the technology of using the modification of a NBS to prepare a Q-body [19]. They first constructed the scFv of an anti-BGP antibody and confirmed that the scFv contained Y91H, W103H, Y36L, and F98L, which are considered to compose the NBS. They then synthesized IBA-C8-TAMRA and connected it to the antibody fragments by ultraviolet irradiation at 254 nm to prepare a Q-body. When the probe was mixed with BGP-C7, the fluorescence intensity increased 9-fold, showing an LOD of 0.56 nM. They also applied this method to polyclonal antihuman serum albumin IgG, and observed antigen-dependent fluorescence increase up to 1.7-fold.

Fukunaga et al. reported another general method for preparing Q-bodies from natural antibodies by selective *N*-terminal alkylation using PEGylated dye aldehyde [20]. This method can simplify the reaction and allow double labeling of the heavy and light chains with the dye to enhance the signal. The alkylamines obtained are expected to reduce steric hindrance and improve the stability of the modified antibody. In addition, it was easy to convert commercial antibodies into fluorescent antibody probes, facilitating the application of this method to the rapid detection of multiple antigens such as biomarkers of acute diseases. The enhanced signal due to double-labeling can be explained by the additional quenching by the dye–dye interaction between the two dyes in H and L chains, which is also called H-dimer formation. Although double-labeled UQ-bodies generally show higher response especially for protein antigens, they sometimes show higher EC50 due to the strong dye–dye interaction. Hence, care must be taken to choose the appropriate dye and linker length to obtain the best performance.

Compared with normal Q-bodies or UQ-bodies, which are classified as fluorogenic probes, ratiometric fluorescent probes are a useful tool for the quantitative detection of target molecules, which are more tolerant to the probe concentration fluctuation. Although first reported by Kajihara et al. [21] and later applied to UQ-body [10], there had been no general and widely used method for the design and synthesis of fluorescent ratiometric probes based on organic dyes. Recently, Yoshikoshi et al. developed a novel scFv-type Q-body derivative based on Förster resonance energy transfer (FRET) and antigen-dependent fluorescence quenching [22]. Fluorescent unnatural amino acids labeled with rhodamine green (rhG) and TAMRA were used as donor and acceptor, respectively, in FRET. In a cell-free translation system, double-labeled single-chain antibodies were synthesized with four bases and amber codons. The resulting double-labeled antibody fragment showed a significant change in the fluorescence ratio upon antigen-binding.

This result was explained by FRET from rhG to TAMRA, but TAMRA was quenched by the Trp residue in the absence of antigen. The binding of antigen could release TAMRA from quenching without changing the FRET efficiency. Such double-labeled antibody fragments will be helpful for diagnosis and imaging applications [10]. Furthermore, we expect the extension of the cell-free synthesis approach to the cell-based approach in near future.

## 4. Fluorescence Quenching Probes for Intracellular Signaling Detection

The Q-body has been applied for detecting not only proteins and peptides but also their modifications. For example, considering that protein phosphorylation is key to intracellular signal transduction, fluorescent biosensors for targeting protein-specific phosphorylation are very useful tools in cell biology and drug screening. Vimentin is the most abundant intermediate filament protein, and its specific serine (Ser) residues are phosphorylated in a cell cycle-dependent manner. Its structure and function are modified by phosphorylation, which affects the biological characteristics of cells. Phosphorylation of vimentin Ser71 (PS71) and vimentin Ser82 (PS82) was detected by Jeong et al. [23]. They found that R6G-labeled Q-bodies showed a better response than TAMRA-labeled probes. Next, they made several probes to detect PS71 and PS82. By optimizing the reaction conditions, the fluorescence intensity of R6G labeled V_H_-V_L_ PS71 probe was increased 4-fold in the presence of excess antigen. In addition, the fluorescence intensity of R6G double-labeled at the *N*-terminus and in the linker of V_L_-V_H_ PS82 probe increased to 6.7-fold when excess antigen peptide was added. Deeper quenching and higher dequenching implied that a quenched dimer called H-dimer was formed between the two dyes.

Fukunaga et al. developed a Q-body to detect phosphotyrosine (pTyr) derivatives [24]. TAMRA-linked unnatural amino acids were incorporated into the *N*- or C-terminus of a scFv antibody against pTyr. Based on fluorescence quenching and antigen-dependent dequenching, the fluorescence of TAMRA labeled scFv was enhanced by the addition of a pTyr-containing peptide. The probe was further fused with enhanced green fluorescent protein (EGFP) to produce double-labeled scFv. In the absence of antigen, EGFP and TAMRA underwent FRET, and the fluorescence of TAMRA was quenched. The antigen binding reduced quenching without a significant change in FRET efficiency. Due to FRET and the quenching effect, the fluorescence ratio of the double-labeled Q-body was enhanced in an antigen-dependent manner, providing a new tool for protein phosphorylation analysis.

The phosphorylation detecting Q-body has advantages of simplicity, rapidity, and high sensitivity. It also has wide application prospects in the field of in vitro diagnosis, drug screening, and imaging.

## 5. Drug Testing

**Figure 3:**
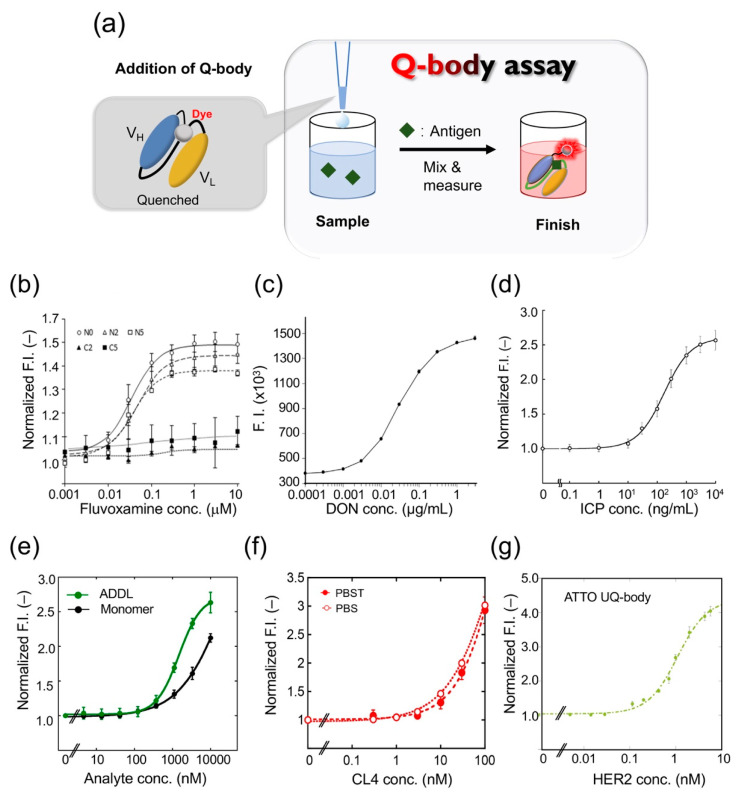
Quenchbody assays for analyte detection. (**a**) Schematic image for Quenchbody assay. (**b**) Detection of fluvoxamine. (**c**) Detection of deoxynivalenol (DON). (**d**) Detection of imidacloprid (ICP). (**e**) Detection of β-amyloid monomer and oligomer (ADDL)s. (**f**) Detection of claudin 4 (CL4) in PBS and PBS containing 0.05% Tween 20 (PBST). (**g**) Detection of human epidermal growth factor receptor 2 (HER2) extracellular domain.

The Q-body assay is convenient because it only includes mixing and measuring (Figure 3a). Therefore, it can be used for rapid drug testing. Drug abuse has become a public health problem worldwide in recent years. Fluvoxamine (FLV), a selective serotonin reuptake inhibitor, is widely used for treating depression and obsessive-compulsive disorder. Although the number of FLV poisoning cases have increased, there is no immunoassay kit for rapid FLV detection [25,26]. Sasao et al. thus created a Q-body for detecting the antidepressant FLV and determined the best conditions for achieving the highest fluorescence intensity [27]. They expressed the recombinant scFv in *E. coli* and refolded the protein from the insoluble fraction by step dialysis. They used linkers of different lengths (0, 2, or 5 repeats of G_3_S units) or different fluorescent dyes (TAMRA-C5, TAMRA-C2, R6G, or ATTO520) to label the *N*-terminal or C-terminal of anti-FLV scFv. Their characteristics were then studied to clarify the best Q-body for FLV detection.

After adding FLV, the Q-body FI of the *N*-terminally Cys-tagged Q-body increased approximately 1.5-fold, whereas the fluorescence intensity of the C-terminally Cys-tagged Q-body did not increase significantly. The maximum fluorescence intensity values of TAMRA-C5, TAMRA-C2, and R6G labeled Q-bodies were 1.3-, 1.2-, and 1.5-fold (Figure 3b), and the EC50 values were 55.2, 23.2, and 224 nM, respectively. The fluorescence intensity of the Q-body labeled with ATTO520 did not change. The PET-mediated quenching effect was found to be more effective with a shorter linker. In conclusion, the N-terminal Q-body with a short linker showed the best effect on improving fluorescence in FLV detection, and R6G was the best fluorescent dye for the system. To determine whether the Q-body was suitable for real samples, the reactivity of the Q-body was tested in serum spiked with FLV. The Q-bodies were found suitable for serum samples after being subjected to deproteinization, to prevent the inherent tryptophan residues in serum proteins from interfering with fluorescence, and showed a dose-dependent effect of FLV.

Tsujikawa et al. have developed a UQ-body assay for detecting marijuana [28]. Cannabis is the most frequently abused drug worldwide. The United Nations Office on Drugs and Crime estimates that 166 million people used cannabis in 2006, accounting for about 4% of the global population aged 15–64 years. The main psychoactive component in cannabis is Δ9-tetrahydrocannabinol (THC), which is the carboxylated form of Δ9-tetrahydrocannabinolic acid (THCA). THCA is decarboxylated to THC during the storage and smoking of marijuana products, and at the gas chromatograph inlet. Fresh marijuana shows a high THCA/THC ratio. The other major components of cannabis are cannabinol (CBN) and cannabidiol (CBD). CBN is a degradation product of tetrahydrocannabinol, which is not found in fresh hemp samples [29].

The detection time of the developed assay was found to be about 80 s, which is shorter than that required for immunochromatography. A simple portable fluorophotometer was designed to measure fluorescence, eliminating the subjectivity of judgment. The experimental results with the UQ-body showed comparability with those of the Duquenois–Levine test. Considering that the sensitivity of the UQ-body test to THCA is low and that to CBN is high, the suitability of the UQ-body test for fresh hemp samples is not good, whereas that for aged hemp samples is good. Thus, heating the samples before testing to decarboxylate THCA to THC may be effective in improving the detectability of fresh marijuana. In this study, sample preparation was carried out in a laboratory environment. However, there are no laboratory instruments such as vortex mixers, centrifuges, and mechanical pipettes at a crime scene. To apply the UQ-body test to drug screening in the field, it is necessary to develop sample preparation equipment suitable for extraction, filtration, and dilution, to facilitate its transportation to and use at crime scenes.

## 6. Detection of Other Small Molecules and Peptides

Deoxynivalenol (DON) is a mycotoxin produced by *Fusarium* spp. Because of their wide geographical distribution, DON has polluted agricultural products including wheat, barley, and corn worldwide [30,31]. To develop a rapid method for determining DON, Yoshinari et al. synthesized the scFv gene using the sequence information of a monoclonal antibody against DON, and prepared a Q-body using a cell-free expression system [32]; when they mixed the Q-body with DON, the fluorescence intensity was increased in a dose-dependent manner (Figure 3c). The detection range was 0.0003 to 3 μg/mL, the coefficient of variation was 7.9% at 0.003 μg/mL, 5.0% at 0.03 μg/mL, and 13.7% at 0.3 μg/mL. When the Q-body was used to detect wheat extract, it showed antigen-dependent fluorescence enhancement with a detection limit of 0.006 μg/mL. To verify the validity of this analytical method, four wheat samples were used for the recovery test with a recovery of 94.9–100.2%. The concentration of DON was then compared in 21 naturally contaminated wheat samples using Q-body, LC-MS/MS, and immunochromatography analysis. LC-MS/MS analysis showed that the contamination level of DON in the samples ranged from 0.001 to 2.68 mg/kg. LC-MS/MS and the Q-body method showed a higher correlation compared with the immunochromatographic kit. These data showed that the developed Q-body could be used to detect DON content in wheat samples, and can be widely used in food safety control.

Rapid detection of environmental pollutants is very important for environmental protection. The pollution of land and water with residues of neonicotinoid insecticides is a serious environmental problem worldwide [33]. However, the traditional method of pesticide residue detection is time-consuming and laborious. To solve this problem, Shao et al. developed a UQ-body for detecting imidacloprid (ICP), a neonicotinoid pesticide [34]. The fluorescent dye TAMRA was used to label the Fab fragment with a specific labeling site. The constructed UQ-body could detect ICP at 10 ng/mL, with a wide working range, even from river water (Figure 3d). The entire process could be completed in a few minutes. Further, the Q-body showed low cross-reactivity with similar compounds that could interfere. As a general technology, this probe has great potential for monitoring neonicotinoid residues in the environment and in food samples.

Alzheimer’s disease (AD) is a neurodegenerative disease involving the formation of senile plaques composed of β-amyloid protein (Aβ), produced by amyloid precursor protein. Among these, Aβ-42 is one of the main peptide forms, which can quickly aggregate and show higher neurotoxicity [35]. Recent studies have shown that Aβ oligomers such as Aβ-derived diffusible ligand (ADDL) are more toxic than fibrils [36]. Rapid detection of Aβ monomers and oligomers is thus of great significance in the diagnosis and treatment of Alzheimer’s disease. Dong et al. developed a UQ-body for the rapid detection of Aβ-42 and ADDL [37]. They expressed the Fab fragment of a humanized anti-Aβ antibody h12a11 in *E. coli*, which preferentially binds Aβ oligomers, and constructed the UQ-body by labeling the Fab fragment with TAMRA or ATTO520. The Q-bodies could detect both ADDL and Aβ-42 monomer, but the sensitivity of the double-labeled UQ-body to ADDL was higher than that of the Aβ monomer (Figure 3e).

## 7. Detection of Proteins

The Q-body is used to detect small molecules and polypeptides as well as proteins and other macromolecules. Claudin (CL) is a membrane protein found in tight junctions, which plays an important role in the establishment of intercellular barriers. It is an important indicator for clinical cancer diagnosis. Jeong et al. constructed a UQ-body based on the Fab fragment of an antibody for detecting and imaging CL overexpressed on tumor cells [38]. They used the variable region genes of anti-CL1 and anti-CL4 antibodies to express recombinant Fab fragments, and selected clones with high affinity to recognize CL4 for preparing UQ-bodies. When the two fluorescent dyes were conjugated to the Fab *N*-termini, the fluorescent signal increased significantly in the presence of purified CL4 at more than nanomolar levels (Figure 3f). Currently, the detection methods for CLs mainly use commercial antibodies through immunohistochemistry techniques such as Western blotting and ELISA. Although these traditional techniques have high specificity and sensitivity, their results sometimes are not accurate due to the high background signal, and are often time-consuming and laborious. Although single-photon emission computed tomography is effectively used as a modality in CL imaging [39], this method has high technical requirements and expensive equipment. As an antibody-based sensor, antigen quantification can be completed within minutes using the UQ-body, whereas most conventional methods require hours or days to obtain the final results. Overall, this study presented results of high sensitivity and convenient detection of CL using a UQ-body in solution.

Influenza is an acute respiratory disease caused by the influenza virus, which has been breaking out globally, with serious consequences to human health. Clinical attention is mainly focused on the emergence of a subtype that causes a pandemic. Influenza A viruses have genes encoding 1–18 hemagglutinin (HA) surface proteins (H1-H18) that promote virus attachment to the host cell surface and its penetration into the cell; they also have genes encoding 1 to 11 neuraminidase (NA) surface proteins (N1-N11) that are related to the release of virus progenies in the host cell [40,41]. Among them, in 2009 the swine influenza A (H1N1) virus led to the first pandemic in the 21st century, which killed about 100,000 people and is still spreading worldwide [42]. Jeong et al. produced a Q-body that can recognize influenza HA using *E. coli* expression and thiol-based fluorescence labeling methods [43]. Using the gene of the anti-HA antibody FI6v3, two types of UQ-body constructs were prepared. As the number of Trp residues (4) of the antibody in V_H_ were more than those in V_L_ (2), the authors attached a Cys tag to the N-terminal of the heavy chain of the Fab expression vector to prepare a single labeled Q-body. In addition, due to the interaction between the dyes, they also constructed a Fab fragment with a Cys label on both *N*-termini of the chain, expecting greater quenching and antigen-dependent fluorescence recovery. These two vectors were used to express Fab derivatives in *E. coli*, and the soluble proteins were recovered and purified by immobilized metal affinity chromatography. Later, they used two fluorescent dyes, TAMRA and ATTO520, which have been proven effective for quenching in anti-BGP Q-bodies, for preparing UQ-bodies. After labeling, the UQ-body was purified to remove free dyes that may produce a background signal. The fluorescence intensity of UQ-body labeled with single TAMRA, double TAMRA, single ATTO520, and double ATTO520 was increased to 1.12 ± 0.01-, 1.81 ± 0.14-, 1.19 ± 0.00- and 4.40 ± 0.12-fold, respectively, and was antigen concentration-dependent. In the presence of antigens, the maximum response of the double-labeled UQ-body was twice as high as that of the single-labeled UQ-body.

The LOD value of the double ATTO520 labeled Q-body with the best fluorescence response was 3.34 ± 0.76 nM. These results showed that the UQ-body can be used as a practical sensor for detecting H1N1 HA [44]. When ATTO520 was used to label the anti-HA Fab produced by bacteria, the fluorescence intensity increased to 4.4-fold in the presence of H1N1 HA. These results indicated that the UQ-body can be used as a fast and simple sensor for detecting influenza A viruses in situ. The method based on *E. coli* for producing Q-bodies can improve yield and cost-effectiveness; therefore, it is expected to be a reliable method for the field detection of the influenza virus.

Human epidermal growth factor receptor 2 (HER2), a receptor tyrosine kinase, usually controls the differentiation and proliferation of mammalian cells [45]. HER2 is also often overexpressed in cancer cells such as stomach cancer and breast cancer, and has become an important biomarker for diagnosis and targeted therapy [46]. The anti-HER2 antibody can block the downstream signal transduction of HER2 positive cells and cause cancer cell death. Dong and Oka et al. constructed a Q-body for detecting HER2 in solution or on the surface of cancer cells with high sensitivity, and then to kill tumor cells [47]. They chose Fab37, an anti-HER2 antibody fragment previously selected from a synthetic binary encoding library by phage display. The most significant feature of this Fab fragment is that its CDR contains a large number of Trp residues [48]. Fab37 with an *N*-terminal cysteine tag on a heavy chain (H-Fab37) and that on a light chain (L-Fab37) were constructed. TAMRA-C6-maleimide and ATTO520-maleimide were used to label H-Fab37 and L-Fab37, respectively. The results showed that the ATTO H-Fab37 UQ-body was the best as it could detect a HER2 concentration as low as 20 pM. The EC50 was calculated to be 0.3 nM, and the response multiple was 4-fold (Figure 3g). The developed UQ-body can detect HER2 in a solution with high sensitivity.

## 8. Applications in Bioimaging and Therapy

**Figure 4:**
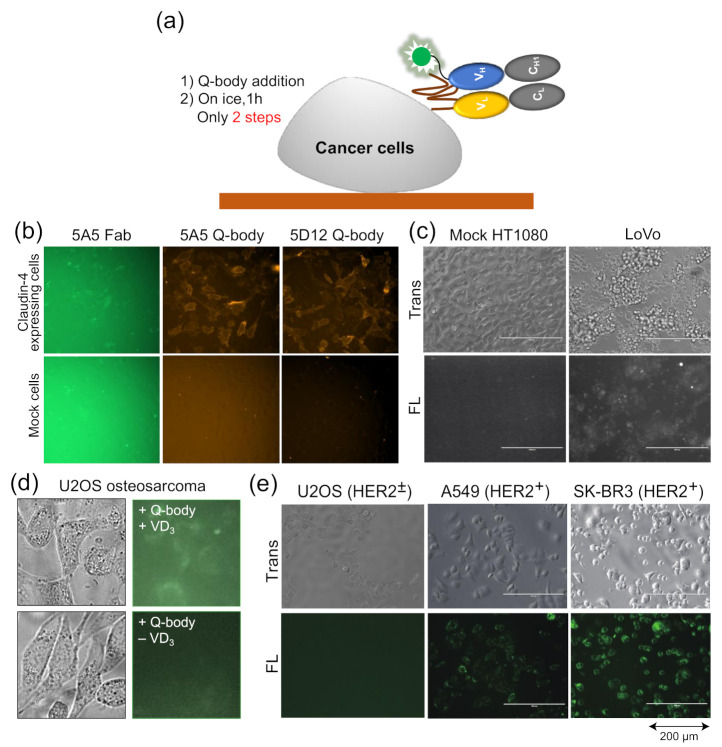
Quenchbodies for imaging cells. (**a**) Bioimaging using Quenchbody; (**b**,**c**) Claudin-4 expressed on the membrane of transfected HT1080 cells (**b**) and LoVo colon cancer cells (**c**). Reprinted with permission from [38]. Copyright 2017 American Chemical Society. (**d**) Bone Gla protein produced by vitamin D3-induced U2OS osteosarcoma; (**e**) HER2 on cancer cells with different HER2 expression levels. Reprinted with permission from [47].

As the Q-body is a fluorescent biosensor probe based on an antibody fragment, it retains the specificity of the antibody, can accurately measure the antigen concentration, and can combine with the antigen specificity of proteins related to cells for fluorescence staining. Traditional fluorescence staining usually requires more than two antibodies and fluorescent probe-labeled antibodies. After the binding of one antibody and antigen, the plate needs to be washed to reduce background. On the other hand, when the Q-body binds the cell surface, its fluorescence intensity increases, and so, it also reduces the fluorescence background, thus eliminating the need for a washing step (Figure 4a).

Dong et al. developed UQ-bodies to image aggregate Aβ with a low background, indicating that UQ-bodies have the potential for rapid biological imaging [37]. When Jeong et al. added the claudin-4 (CL4) UQ-body to CL4-positive transfected cells, they observed a clear fluorescence signal with a low background (Figure 4b) [38]. The same UQ-body could also be used to image antigens on the surface of LoVo colon cancer cells that express CL4, but not on control HT1080 cells (Figure 4c). The BGP Q-body was used to determine the BGP concentration in samples until Abe et al. used it to detect BGP secretion in cells. U2OS osteosarcoma secretes BGP upon addition of vitamin D3 (VD_3_) to induce differentiation into osteoblast-like cells. When UQ-bodies were added to cell cultures with VD_3_, BGP production on the plasma membrane was clearly observed by fluorescence microscopy (Figure 4d) [10]. HER2 is a tumor biomarker overexpressed in many cancer cells, including A549 and SK-BR3 cells. The cells were clearly visualized using the HER2 UQ-body, as shown in Figure 4e. These studies showed that the Q-body can be used as a tool to detect the surface or secreted markers of cancer cells, at least in vitro.

In addition to imaging, they used UQ-body as a carrier to deliver siRNA by adding nine continuous arginine sequences (9R) to its C-terminal region. To explore the therapeutic effect of HER2 positive cancer cells as an RNAi delivery platform, Polo like kinase 1 (Plk1) was selected as the target protein to achieve efficient killing by siRNA in tumor cells. Plk1 siRNA delivered by UQ-body-9R can effectively kill HER2-positive cancer cells. This new method can detect and image HER2 antigen conveniently and sensitively, and can effectively kill HER2-positive cancer cells using UQ-bodies combined with siRNA attached to the C-terminal 9R sequence. Thus, the Q-body can also be used as a delivery carrier to deliver targeted therapeutic drugs against serious diseases, especially cancer.

## 9. Application to Nanobodies

Q-bodies can detect many target molecules rapidly simply by mixing them with a sample. However, their applicability to camelid heavy-chain antibody variable domain (V_HH_ or nanobody) has been elusive. Recently, nanobodies have received considerable attention owing to their small size (~14 kd) compared to scFv or Fab, and their high stability and resultant high production yield when produced in *E. coli* [49]. Since their first report, many examples of nanobodies that bind antigens specifically and strongly have been reported. Although it has been considered difficult to raise good small-molecule binders compared with protein binders, several examples of anti-hapten nanobodies have also been reported [50].

**Figure 5:**
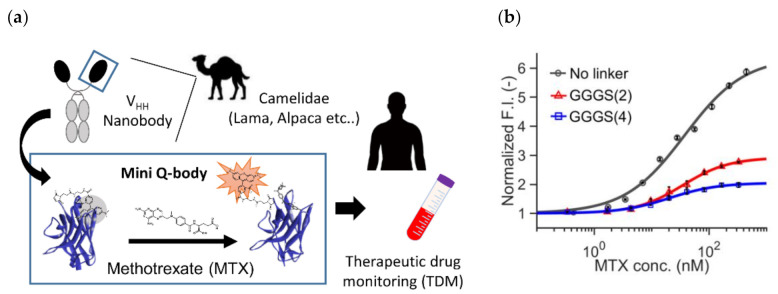
Mini Q-body (**a**) The scheme of methotrexate (MTX) therapeutic drug monitoring (TDM) based on mini Q-body. (**b**) Dose-response curves of three mini Q-bodies with a linker at different lengths. Reprinted with permission from [51]. Copyright 2020 American Chemical Society.

Recently, Inoue et al. reported the successful construction of a Q-body that can detect a small molecule methotrexate (MTX) using a specific V_HH_ fragment [51]. MTX is known as a specific inhibitor of mammalian dihydroforate reductase (DHFR), which participates in nucleic acid metabolism. Hence, it has been used as a chemotherapeutic agent against cancer. However, as normal cells also have DHFR, higher doses might lead to the risk of side effects. In addition, MTX is known to have immunosuppressive activity, and its effective dose is low. Hence, monitoring its blood concentration (therapeutic drug monitoring, TDM) is considered very important (Figure 5a).

To this end, they constructed three types of “mini” Q-bodies labeled with a dye at their *N*-terminal region, with different lengths of linker between the Cys-tag and V_HH_ *N*-terminus. After the expression of the protein in *E. coli* and its purification, the nanobodies were labeled with several types of dyes, and it the mini Q-body labeled with TAMRA without the linker manifested good antigen-dependent fluorescence of more than 6-fold (Figure 5b). Moreover, the LODs in the buffer and in 50% human serum were 0.56 nM and 1.72 nM, respectively, which were low enough for TDM. According to the crystal structure of the nanobody with bound MTX (PDB 3QXV), the antigen MTX is mainly recognized by CDR1, and from the analysis of mutants, Trp34 in CDR1 was found to play a major role in the quenching of the dye. Hence, the binding mode and existence of neighboring Trp at the antigen-binding site might be key factors in creating such good mini Q-bodies.

## 10. Q-Probe

**Figure 6:**
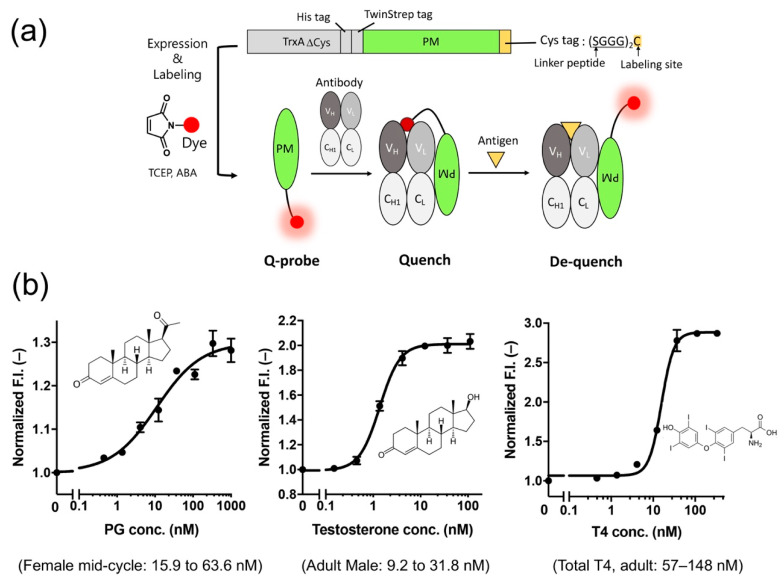
(**a**) Scheme of the structure and use of the PM Q-probe. (**b**) Results of small molecule detection using the IgG/PM Q-probe complex. The normal concentration range in blood for each dose-response is shown in parentheses. Modified with permission from [52].

Recently, we developed a probe (PM Q-probe) that can easily convert many antibodies, including commercially available ones, into fluorescent biosensors using a recently discovered antibody-binding protein Protein M (PM) [52]. The PM, isolated from human mycoplasma, was found to bind strongly (*K*_d_~low nM) to a wide range of antibodies at the light chain, and is considered to disrupt the immune system by structurally blocking the binding of antibodies to (protein) antigens [53]. We found that the C-terminal region of PM is close to the antigen-binding site of the antibody in the solved structures, and hypothesized that binding of smaller antigens such as peptides and haptens is not interfered by the PM upon binding. Thus, we constructed a probe containing a dye introduced into the C-terminus of PM via a short linker and named it PM Q-probe (Figure 6a). This Q-probe was found to have properties similar to Q-bodies, as its fluorescence was quenched by 50% when mixed with osteocalcin-recognizing antibody Fab fragments or full-length IgG antibodies, which are bone disease markers that were used as models, and its fluorescence was restored when osteocalcin or its C-terminal fragment was added as the antigen. Interestingly, the complex with IgG showed higher antigen detection sensitivity than the recombinantly constructed Fab. It is worth noting that the full-length osteocalcin (49 aa) was also detected by Q-probe/Fab complex with similar sensitivity to the scFv BGP Q-body, suggesting that not only small molecule but also small proteins are detectable by PM Q-probe.

Next, we measured the concentrations of many low-molecular-weight biomarkers such as progesterone, testosterone, and thyroid hormone T4, which are used in blood diagnostics, using their respective IgG antibodies including commercially available ones. As a result, we could quantify their concentrations in the buffer with the sensitivity covering the working range in the blood of healthy subjects. We also succeeded in determining the concentration of T4 extracted from serum using ethanol. In other words, we could determine the total T4 concentration, including T4 bound to proteins, in the blood (Figure 6b). However, direct measurement from serum samples was difficult because of the dissociation of Q-probe/IgG complexes caused by antibodies present in the serum.

Therefore, using four hybridomas secreting monoclonal antibodies that recognize cortisol, a stress hormone, as the starting material, we selected a promising clone using a Q-probe and constructed a cortisol Q-body by genetic engineering of the genes from antibody-producing cells. The constructed Q-body showed a fluorescence response similar to that of the Q-probe/IgG complex. Furthermore, the performance of the Q-body in 50% serum was comparable to or even better than that of the Q-probe in buffer. These results indicate that Q-probes can be used to select antibodies suitable for Q-bodies, and that the resulting Q-bodies can be used for the direct measurement of antigens in serum. These results clearly suggest that this technology will expand the application range of Q-bodies and facilitate the timely construction of Q-bodies or a Q-probe/IgG complex that detects various target molecules with high response and sensitivity.

## 11. Prospects of Q-Body Technology

As a rapid fluorescent homogeneous immunoassay probe, Q-body can be used to detect a series of substances, such as small molecules, peptides, and proteins. Q-bodies labeled with a variety of dyes may be used to simultaneously detect a variety of substances in the same sample in future.

The merits of Q-body technology over other immunoassays can be summarized as follows.

(1) Simplicity. No necessity of other reagents and steps, especially washing.
(2) Quickness of the assay. The assay time only depends on the binding kinetics of antibody.
(3) Allows noncompetitive detection for small molecules. Signal increases, unlike competitive assays. Competition of the dye and antigen only happens inside the Q-body, hence it ensures lower experimental error. In addition, preparation of antigen-dye (or carrier) conjugate is not necessary.

We also can make various types of Q-bodies using scFv, Fab, IgG and nanobody. Each format has merit, for example, scFv and nanobody are easy to express, and Fab and IgG can be more sensitive, especially when introduced with multiple dyes. On the other hand, scFv is not very stable and Fab manifests low yield when expressed. IgG protein can be chemically modified easily, but its engineering is just its beginning. To overcome the previous limitations of Q-bodies, e.g., the need considerable genetic engineering to clone the antibody gene and testing for several clones with different combinations of dye and linkers, fluorescence-labeled antibody binding proteins (Quench probes, Q-probes) have been developed, which can easily convert the available antibody protein to a Q-body.

At present, semirational design of Q-body can be performed as follows:

For detecting small antigens:

1. By using a PM Q-probe, find a suitable antibody that shows deep quenching upon binding and high dequenching upon subsequent antigen addition.
2. Clone V_H_/V_L_ genes to construct single- (or double-) labeled Q-body (scFv- or Fab-type).
3. Optimize the dye position (H or L chain *N*-terminus) and linker length.

For detecting proteins:

1. Prepare candidate antibody genes with a sufficient number of Trp residues in its V_H_/V_L_ region.
2. Make double-labeled Fab-type Q-bodies with relatively short linker peptides.
3. Perform antigen detection assay to find the best clone.

Another potentially promising approach is molecular evolution, construction of a suitable antibody library (with Trp-rich CDR as exemplified in Fab37), and selection based on fluorescent responses. We envisage that the combination of appropriate display technologies such as phage display and yeast display will give us an efficient and timely selection of good recombinant Q-bodies. Yet another potential approach in the near future is the use of molecular dynamics simulation to design high-performance Q-bodies in silico [54].

Thus, the Q-body has important theoretical significance and broad application prospects in biosensing and biological imaging as a new type of immunosensor.

## Data Availability

Not applicable.

## Abbreviations

nine arginine sequences (9R); 3-(6-acetylnaphthalen-2-ylamino)-2-aminopropanoic acid (Anap); β-amyloid protein (Aβ); Aβ derived diffusible ligand (ADDL); background/free (B/F); C-terminal 7 residue peptide of bone Gla protein (BGP-C7); bisphenol A (BPA); cannabidiol (CBD); cannabinol (CBN); complementarity determining region (CDR); Claudin (CL); dibenzocyclooctyne (DBCO); dihydroforate reductase (DHFR); deoxynivalenol (DON); enhanced green fluorescent protein (EGFP); epidermal growth factor receptor (EGFR); enzyme-linked immunosorbent assay (ELISA); gas chromatography coupled with mass spectrometry (GC/MS); antigen-binding fragment of antibody (Fab); heavy chain of Fab fragment (Fd); antibody variable region (Fv; VH + VL); fluvoxamine (FLV); fluorescence polarization (FP); Förster (fluorescence) resonance energy transfer (FRET); guanidine hydrochloride (GdnHCl); hemagglutinin (HA); human epidermal growth factor receptor 2 (HER2); human immunodeficiency virus (HIV); indole-3-butyric acid (IBA); imidacloprid (ICP); liquid chromatography coupled with mass spectrometry (LC/MS); limit of detection (LOD); methotrexate (MTX); neuraminidase (NA); nucleotide binding site (NBS); phosphate buffered saline (PBS); PBS containing 0.05% Tween 20 (PBST); *p*-phenylenediamine (*p*-PDA); photoinduced electron transfer (PET); polo like kinase1 (Plk1); Protein M (PM); phosphotyrosine (pTyr); Quenchbody (Q-body); Quenchprobe (Q-probe); rhodamine 6G (R6G); n-methylpyridine-4-aldehydebenzenesulfonates (Rapoport’s salt, RS); rhodamine green (rhG); single-chain variable region (scFv); serine (Ser); scaffold conjugated to environment-sensitive fluorophore (SuCESsFul); tetramethyl rhodamine (TAMRA); therapeutic drug monitoring (TDM); tetrahydrocannabinol (THC); tetrahydrocannabinolic acid (THCA); tryptophan (Trp); unnatural amino acid (UAA); ultra Q-body (UQ-body); vitamin D3 (VD3); variable region of heavy chain (V_H_); camelid heavy-chain antibody variable domain (V_HH_); variable region of light chain (V_L_).
